# Artificial Menopause

**Published:** 1899-10-21

**Authors:** 


### ARTIFICIAL MENOPAUSE.

Speaking at Portsmouth, Mr. Bland Sutton drew a comparison between the natural menopause and that artificially produced by removal of the ovaries or uterus. The normal menopause, he says, may be defined as the cessation of menstruation in consequence of the natural atrophy of the secreting tissue of the ovaries, and it is declared not only by the cessation of the monthly loss of blood from the uterus, but by the peculiar cutaneous phenomenon known as "flushing," and by other nervous symptoms. When both ovaries are completely removed from a sexually perfect woman the result is an abiding amenorrhœa, and the individual exhibits these other peculiarities characteristic of the menopause, so that the result of this operation is truly an artificial menopause. When, however, the uterus is removed and the ovaries are left, although amenorrhœa and sterility are produced, this cannot be properly called an artificial menopause. It is, in fact, not a menopause at all in the true sense of the term; and it is this consideration which has led Mr. Bland Sutton, wherever it was practicable and prudent, in performing hysterectomy, to spare at least one ovary, and to remove the uterus in many grave conditions in preference to the sacrifice of both ovaries. The relation of these forms of artificial menopause to marriage are matters of some social importance, in regard to which Mr. Bland Sutton says, "When I am called upon to give counsel in regard to it, I feel that if both ovaries have been removed there is little prospect of a happy union, and generally advise against marriage. On the other hand, if a woman has had her uterus removed and possesses at least one ovary, she may be regarded as nubile, if the intended husband clearly understands that the marriage will be sterile."

